# Influence of Electron Beam Irradiation and RPMI Immersion on the Development of Magnesium-Doped Hydroxyapatite/Chitosan Composite Bioactive Layers for Biomedical Applications

**DOI:** 10.3390/polym17040533

**Published:** 2025-02-18

**Authors:** Andreea Groza, Maria E. Hurjui, Sasa A. Yehia-Alexe, Cornel Staicu, Coralia Bleotu, Simona L. Iconaru, Carmen S. Ciobanu, Liliana Ghegoiu, Daniela Predoi

**Affiliations:** 1 National Institute for Lasers, Plasma and Radiation Physics, 077125 Măgurele, Romania; maria.zarif@inflpr.ro (M.E.H.); sasa.yehia@inflpr.ro (S.A.Y.-A.); cornel.staicu@inflpr.ro (C.S.); 2 Faculty of Chemical Engineering and Biotechnologies, University Politehnica of Bucharest, 011061 Bucharest, Romania; 3 Faculty of Physics, University of Bucharest, 077125 Măgurele, Romania; 4 Department of Cellular and Molecular Pathology, Stefan S. Nicolau Institute of Virology, Romanian Academy, 030304 Bucharest, Romania; cbleotu@yahoo.com (C.B.); simonaiconaru@gmail.com (S.L.I.); 5 Research Institute of the University of Bucharest (ICUB), University of Bucharest, 060023 Bucharest, Romania; 6 The Academy of Romanian Scientist, 050711 Bucharest, Romania; 7 National Institute of Materials Physics, 077125 Magurele, Romania; ciobanu_carmen83@yahoo.com (C.S.C.); ghegoiuliliana@gmail.com (L.G.); dpredoi@gmail.com (D.P.); 8 Department of Mechanics, University Politehnica of Bucharest, 060042 Bucharest, Romania

**Keywords:** magnesium-doped hydroxyapatite/chitosan coatings, radio-frequency magnetron sputtering deposition technique, electron beam irradiation, MG63 cell line, MTT assay

## Abstract

Magnesium-doped hydroxyapatite/chitosan composite coatings produced by the radio-frequency magnetron sputtering technique were exposed to 5 MeV electron beams of 8 and 30 Gy radiation doses in a linear electron accelerator. The surfaces of unirradiated layers are smooth, while the irradiated ones exhibit nano-structures with sizes that increase from 60 nm at a 8 Gy dose to 200 nm at a 30 Gy dose. Young’s modulus and the stiffness of the layers decrease from 58.9 GPa and 10 µN/nm to 5 GPa and 2.2 µN/nm, respectively, when the radiation doses are increased from 0 to 30 Gy. These data suggest the diminishing of the contribution of the chitosan to the elasticity of the magnesium-doped hydroxyapatite/chitosan composite layers after electron beam irradiation. The biological capabilities of the coatings were assessed before and after their immersion in RPMI-1640 cell culture medium for 7 and 14 days, respectively, and further cultured with a MG63 cell line (ATCC CRL1427) in Dulbecco’s Modified Eagle Medium supplemented with fetal bovine serum, penicillin–streptomycin, and L-glutamine. Thus, 1 µm spherical structures were developed on the surfaces of the layers exposed to a 30 Gy radiation dose and immersed for 14 days in the RPMI-1640 biological medium. The molecular structures of all the RPMI-1640 immersed samples were modified by the growth of a carbonated hydroxyapatite layer characterized by a B-type substitution, as Fourier Transform Infrared Spectroscopy revealed. The biological assay proved the increased biocompatibility of the layers kept in RPMI-1640 medium and enhanced MG63 cell attachment and proliferation. Atomic force microscopy analysis indicated the elongated fibroblastic cell morphology of MG63 cells with minor alteration at 30 Gy irradiation doses as a result of layer biocompatibility modifications.

## 1. Introduction

Calcium phosphates have been intensively studied over the years due to their potential for bone regeneration [1]. Among them, many investigations focused on synthesis of hydroxyapatite (HAp) as bulk materials, powders, or layers, as it is the main inorganic compound in bones [1]. In addition to the chemical elements contained in synthetic HAp, Ca_10_(PO_4_)_6_(OH)_2_ samples in bones, enamel, or dentin, several ions are also constituents. For instance, carbonate, sodium, or magnesium have been reported to have weight percentages of 7.4, 0.9, and 0.72 in bones and 5.6, 0.6, and 1.26 in dentin [1]. Therefore, anionic or cationic substitutions in HAp structures have been considered optimal solutions to mimic the bone composition and to obtain improved biological performances. Anionic substitutions consist of the replacement of the hydroxyl group with monovalent ions such as Cl^−^ or F^−^ or the replacement of the phosphate group with bivalent ions such as HPO_4_^2−^, SO_4_^2−^, or CO_3_^2−^. Cationic substitutions involve the replacement of calcium ions by monovalent (e.g., Na^+^ or Ag^+^), bivalent (e.g., Sr^2+^, Zn^2+^, or Mg^2+^), or multivalent ions [2]. Biological apatite present in bones or teeth and the apatite found in natural rocks contain carbonate groups in different percentages [3]. In the human body, carbonate groups could substitute in the hydroxyapatite structure and could lead to an increased resorption and regeneration of bones.

Magnesium has been reported to be one of the most abundant cations in the human body [4] and 60% is stored in bones [5]. It is involved in bone growth, regeneration, and mineralization and influences the activity of osteoblast and osteoclast cells [4,5]. It has been reported that magnesium deficiency significantly affects bone remodeling. As a direct effect, a low Mg concentration decreases the number of osteoblasts, (the cell bones involved in bone formation), increases the number of osteoclasts, (the cell bones involved in bone resorption), and reduces bone stiffness. As an indirect effect, Mg deficiency affects the homeostasis of the parathyroid hormone and vitamin D, which results in a decrease in bone formation. Moreover, Mg deficiency also induces inflammation, which increases bone resorption [6]. Magnesium also improves the antibacterial effects against *C. albicans*, *S. aureus*, *P. aeruginosa*, and *E. coli* [2], which are usually associated with implant infections. Therefore, magnesium is a great candidate for the substitution of Ca in the HAp structure, especially for applications involving implant coatings.

In order to mimic the organic part of bones, which is mainly composed of type I collagen but also of glycosaminoglycans, proteoglycans, and glycoprotein, chitosan (Cs) is a viable option due to the similarity between the polysaccharide backbone of Cs and glycosaminoglycans [7,8]. Chitosan is a biopolymer derived from chitin through deacetylation [8]. It is biocompatible and biodegradable, which is essential for implant coatings, while the foreign-body response and fibrous encapsulation are reduced [9]. It is also known for its antimicrobial action [10].

According to previous studies [11,12,13], cell culture medium (e.g., DMEM, RPMI, PBS, etc.) could be used as an alternative to conventional simulated body fluid (SBF) to obtain valuable information about a biomaterial’s interactions under simulated physiological conditions. RPMI medium contains nutrients and ions that mimic body fluids [14], providing a relevant environment to study in vitro biomaterial behavior. With the aid of RPMI medium, relevant information regarding biological properties such as cytocompatibility, cell adhesion, proliferation, and differentiation could be obtained in vitro. Therefore, immersing biomaterials in RPMI medium for various time intervals could provide a comparative analysis of the sample’s performance over various periods, helping to understand its behavior and suitability for medical uses [11,12,13,14]. Being a nutrient-rich culture medium, RPMI provides a favorable environment for the growth and development of different cell types, including osteoblasts and other bone-related cells, helping with the evaluation of cellular responses to different biomaterials [12,15]. Due to its extremely well-balanced composition, the use of RPMI in assessing biological properties of materials ensures that the observed effects are primarily attributable to the tested material rather than to external nutrient deficiencies, therefore providing valuable insights regarding the tested material’s compatibility for medical applications.

The conventional cancer treatments are surgery, chemotherapy, and/or radiotherapy [16]. Two types of radiation therapy exist: external, in which the radiation source is not in contact with the body part to be treated, and internal, in which the radiation source is placed inside or beside the tumor [17]. The types of radiation used in radiotherapy are photon radiations, such as X-rays and gamma-rays, and particle radiations, such as electron, proton, and neutron beams [18]. Electron beam therapy has the advantages of a high surface dose and a rapid fall-off of the dose, with electron energies of 4–22 MeV [19]. Due to this, electron beam therapy is usually used to treat tumors that are closer to the surface of the body [20]. However, intraoperative electron radiation therapy (IOeRT) can be used to increase the efficiency of local treatments. Among the tissues that can be affected by IOeRT, bone is also mentioned [21]. As a consequence, the coatings deposited on bone implants can also be affected. For the treatment of the metastatic skeletal pain, clinical research reveals the efficiency of an 8 Gy single-fraction radiotherapy procedure [22]. On the other hand, a 30 Gy total dose is also used in painful bone metastases [23]. There are also recent studies that present the superiority for bone remineralization of the 30 Gy treatment in osteolytic bone metastases [24].

In this regard, our present study offers novel insights on the biological, mechanical, and physicochemical modifications produced by the electron beam irradiation of magnesium-doped hydroxyapatite/chitosan composite layers (MgHApCs) deposited on Si substrates by the radio-frequency magnetron sputtering (RF-MS) technique. Two ionizing radiation doses of 8 and 30 Gy were selected for this study due to their practical use in radiotherapy treatments. In our previous study [25], we evidenced the morphological and physicochemical modifications generated by the irradiation of such layers at 2 and 50 Gy. However, the influence of the electron beam irradiations in relation to the modifications that could appear inside the body were not studied. Some early results [26] revealed that Dulbecco’s Modified Eagle Medium (DMEM) medium influences the physicochemical properties of similar composite coatings, Mg-doped HAp/Cs, synthetized by the spin-coating technique.

Therefore, in the present study, in order to mimic the body environment, the unirradiated and 8 and 30 Gy irradiated coatings were maintained in RPMI-1640 cell culture medium (Roswell Park Memorial Institute Medium) for 7 and 14 days for increasing their biocompatibility. The performed investigations evidence the modifications produced. To our knowledge, no data have been reported previously about the influence of the RPMI-1640 cell culture medium on RF-MS deposited MgHApCs composite layers exposed to electron beams. Further, irradiated and unirradiated samples were introduced in DMEM that also contained fetal bovine serum (FBS), penicillin–streptomycin, and L-glutamine for culture of MG63 cells.

This study uniquely investigates the combined effects of RPMI immersion and electron beam irradiation on the physicochemical and biological features of magnesium-doped hydroxyapatite/chitosan composite coatings. This research provides a comprehensive physicochemical, mechanical, and biological evaluation, highlighting its potential for orthopedic and dental implants.

## 2. Materials and Methods

### 2.1. Materials

The magnesium-doped hydroxyapatite in chitosan matrix powders (MgHApCs) were prepared using the following reagents: magnesium nitrate hexahydrate (Mg(NO_3_)_2_·6H_2_O, 99.97%, Alfa Aesar, Kandel, Germany), calcium nitrate tetrahydrate (Ca(NO_3_)_2_·4H_2_O, ≥99.0%, Sigma Aldrich, St. Louis, MO, USA), diammonium hydrogen phosphate ((NH_4_)_2_HPO_4_, ≥99.0%, Sigma Aldrich, St. Louis, MO, USA), chitosan in various proportion of N-acetyl glucosamine and glucosamine units (C_12_H_24_N_2_O_9_; Sigma-Aldrich, St. Louis, MO, USA; CAS number: 9012-76-4; Molecular weight: 50,000–190,000 Da; Source: shrimp shells; Deacetylation degree: 75–85%), and CH_3_COOH (acetic acid, Sigma Aldrich, St. Louis, MO, USA, ≥99.7%). During the synthesis process, deionized water was used for dissolving the precursors.

Silicon plates with dimensions of 10 × 10 × 1 mm^3^ and polished mirror-like surfaces were used as substrates for the deposition of MgHApCs layers generated by the magnetron sputtering technique.

### 2.2. Synthesis Technique

#### Synthesis of Magnesium-Doped Hydroxyapatite in Chitosan Matrix Powders (MgHApCs)

For the development of MgHApCs powders (x_Mg_ = 0.07), we used an adapted coprecipitation method [27,28]. In brief, for synthesis, the (Ca + Mg)/P ratio was set at 1.67. The MgHApCs synthesis involved dissolving stoichiometric quantities of magnesium and calcium precursors in a beaker in order to obtain 300 mL of aqueous solution, followed by its dropwise addition to a phosphate aqueous solution (300 mL) [27,28]. The mixture ((Ca + Mg)/P = 1.67) was stirred for 12 h at 80 °C, then centrifuged and washed with deionized water four times [27,28]. The chitosan was dissolved in an acetic acid solution (1 wt.%). Finally, the precipitate was redispersed in a 2% chitosan solution, stirred continuously for 4 h at 100 °C, and dried at 80 °C in order to obtain MgHApCs powders.

Afterwards, the powder was pressed mechanically, and we prepared the sputtering target to be positioned inside the magnetron head. The sputtering target was a disc with the dimensions of a 50 mm diameter and 4 mm thickness.

### 2.3. Deposition and Electron Beam Irradiation Techniques

#### 2.3.1. Synthesis of MgHApCs Layers in Radio-Frequency Magnetron Sputtering Discharge

Magnesium-doped hydroxyapatite/chitosan composite coatings on Si substrates were produced via radio-frequency magnetron sputtering discharge and had working parameters as follows: Ar working gas at the 5 × 10^−3^ mbar pressure (10^−5^ mbar base pressure) and 2 mL_n_/min gas flow; 70 W rf power; 6 h deposition time; 4 cm distance between the substrate and the magnetron head. The thickness of the layers was calculated at approximatively ~300 nm by the in situ measurement of the deposition rate in the central part of the substrate holder. More details on the synthesis procedure can be found in [25].

#### 2.3.2. Electron Beam Irradiation of MgHApCs Layers

All the samples were irradiated with electron beams produced in a Siemens (Munchen, Germany) MEVATRON Primus clinical linear accelerator. The surface of irradiation of the samples placed in front of the electron beams was 20 × 20 cm^2^ at a 100 cm distance from the electron beam focal point. The electron beam energy was 5 MeV and the absorbed dose rate was of 3.00 Gy/min, with an error of 5%. The total absorbed doses in the samples, in these conditions, were 8 Gy or 30 Gy. These parameters were measured and calculated prior to sample irradiation, in accordance with the available medical radiation therapy protocols [29].

Following their exposure to electron beams, we selected samples indexed as MgHApCs, MgHApCs-8Gy, and MgHApCs-30Gy that had been immersed in RPMI-1640 medium (Sigma Aldrich, St. Louis, MO, USA) for 7 and 14 days at 37 ± 0.5 °C in an incubator (GFL 4010, GFL Gesellschaft für Labortechnik mbH, Burgwedel, Germany). The RPMI medium was changed daily. After each immersion period (7 and 14 days), the layers were rinsed with bidistilled water and stored in a desiccator. The layers immersed for 7 days were designated as MgHApCs-7D, MgHApCs-8Gy-7D, and MgHApCs-30Gy-7D, while those immersed for 14 days were labeled as MgHApCs-14D, MgHApCs-8Gy-14D, and MgHApCs-30Gy-14D.

### 2.4. Characterization Techniques

The arithmetic mean deviations of the roughness profiles Ra of the Si substrates and all MgHApCs layers were measured with a Mahr Perthometer S2 instrument (Göttingen, Germany). The elastic modulus and the stiffness of the samples were measured by using a Hysitron TI 950 TriboIndenter (Bruker, Eden Prairie, MN, USA) at a load force of 100 µN and a displacement range of 130 µm. The mean value for the indentation depth of the layers was 20 nm.

The acquisition of the attenuated total reflection (ATR) IR spectra of the MgHApCs layers exposed and unexposed to ionizing radiation, before and after preservation in RPMI- 1640 culture cell medium for 7 and 14 days, was performed using a Perkin Elmer SP-100 Fourier Transform Infrared (FTIR) spectrometer (Waltham, MA, USA) with a 4 cm^−1^ resolution. The ATR unit attached to the spectrometer had an KRS-5 crystal, which allowed the recording of the spectra in the 4000–400 cm^−1^ range. The ATR unit was equipped with metallic masks positioned in front of KRS-5 crystal to allow proper analysis of liquid, powder, or solid samples. By using such accessories, we recorded the FTIR spectra of a 10 µL liquid drop of RPMI-1640 medium and all the layers.

Scanning electron microscopy (SEM) investigations of all the samples were carried out by using a ThermoFisher (Waltham, MA, USA) Apreo S scanning electron microscope working in both high- and low-vacuum modes. The microscope had an attached EDAX Inc. SiLi detector (Waltham, MA, USA). Energy-dispersive spectroscopy (EDS), as well as the SEM investigation, were conducted at an acceleration voltage of 10 kV. Magnifications varied between 500× and 40,000×. The 3D surface plots of the SEM images were produced by using Image J software (ImageJ 1.51j8, National Institutes of Health, Bethesda, MD, USA).

The biocompatibilities of the MgHApCs layers unirradiated and irradiated using 8 and 30 Gy doses before and after being immersed in RPMI medium for 7 and 14 days were evaluated with the aid of the MG63 cell line (ATCC CRL1427). The experiments were performed as previously described by Iconaru et al. [30]. For this purpose, the MG63 cells were cultured in Dulbecco’s Modified Eagle Medium (DMEM) supplemented with fetal bovine serum (FBS), penicillin–streptomycin, and L-glutamine. The cultures were maintained at 37 °C in a humidified atmosphere with 5% CO_2_. The MgHApCs layers were placed in 24-well plates, with one pellet per well, and MG63 cells were seeded at a density of 1 × 10⁵ cells/well. Cell viability was assessed after 24 and 48 h of incubation with the aid of the MTT reduction assay (3-(4,5-dimethylthiazol-2-yl)-2,5-diphenyltetrazolium bromide). After each incubation period, the cells were washed using phosphate-buffered saline (PBS) and then incubated with 1 mg/mL of MTT solution and kept in the dark for 3 h. The cell viability was quantified from the optical density measured at 595 nm using a TECAN spectrophotometer (Mannedorf, Switzerland) as a percentage relative to the control sample (100% viability). After 48 h of incubation, the adhered cells on the layer surfaces were fixed using glutaraldehyde, dehydrated with graded ethanol, and air-dried. The prepared samples were analyzed with the aid of atomic force microscopy (AFM) to investigate cell adherence, development, and interaction with the layer surfaces. The biological assays were carried out in triplicate, and the results were depicted graphically as mean ± SD. The statistical analysis was performed using Microsoft Excel (version 2021) using ordinary one-way ANOVA for data analysis, with the significance level set at *p* < 0.05.

## 3. Results and Discussions

### 3.1. Mechanical Tests

The influence of the chitosan embedded in MgHApCs layers exposed and unexposed to electron beams has been investigated firstly by mechanical tests. The results are presented in Table 1.

The arithmetic mean deviation Ra of the roughness profile values increases, possibly because of the patterning of the layer surfaces due to polymer shrinkage. The R_a_ of the Si substrate is 0.008 µm.

Young’s modulus is an expression of the elasticity of a material being defined as the ratio between the stress/strain, where the stress is the force on an area unit applied to a material and the strain is the deformation in the length of the material. The measured values of Young’s modulus decrease as the radiation dose increases. Consecutively, the measured values of the stiffness decrease with the increase in the radiation dose.

### 3.2. FTIR Analysis

**Figure 1:**
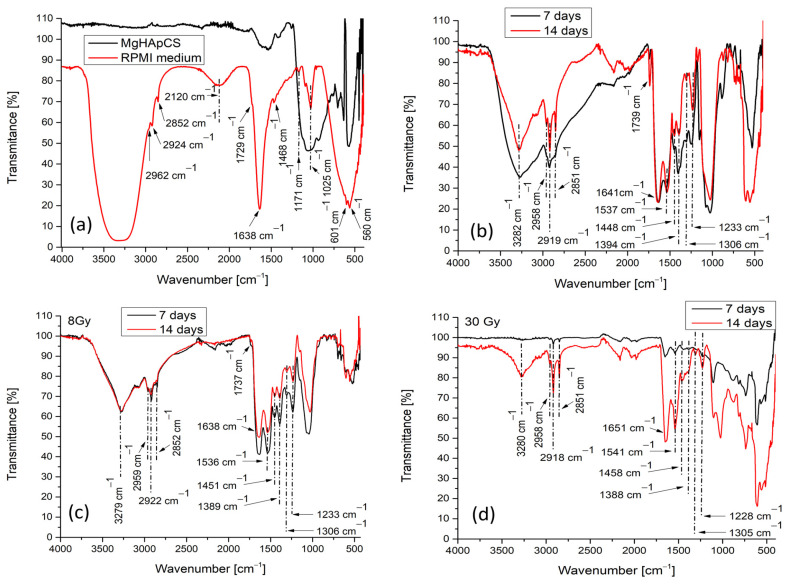
FTIR spectra: (**a**) MgHApCs layers (black line) and RPMI medium (red line); (**b**) MgHApCs-7D (black line) and MgHApCs-14D (red line) samples; (**c**) MgHApCs-8 Gy-7D (black line) and MgHApCs-8Gy-14D (red line) samples; (**d**) MgHApCs-30 Gy-7D (black line) and MgHApCs-30 Gy-14D (red line) samples.

The molecular characteristics of the MgHApCs layers exposed and unexposed to electron beams are evaluated before and after their insertion and held for a maximum period of 2 weeks in RPMI-1640 cell culture medium (see Figure 1a–d). The RPMI-1640 medium contained sodium bicarbonate (NaHCO_3_), glucose (C_6_H_12_O_6_), phenol red (C_19_H_14_O_5_S), and the following ions Na^+^, K^+^, Ca^2+^, Mg^2+^, Cl^−^, PO_4_^2−^, HCO_3_^−^, and SO_4_^2−^ [14].

The active IR bands of P-O and O-P-O vibrations in [PO_4_]^3−^ groups manifest their presence in a compound mainly in the 1200–400 cm^−1^ range and are assigned to the following characteristic vibrational modes: ν_3_—1200–900 cm^−1^; ν_1_—960 cm^−1^; ν_4_—630–500 cm^−1^; ν_2_—470 cm^−1^ [31].

The IR spectrum of the MgHApCs layer, as produced by magnetron sputtering discharge (see Figure 1a black line), indicated a broad band with two maxima at 1025 and 930 cm^−1^ assigned to P-O asymmetric and symmetric stretching vibrations in [PO_4_]^3−^ groups of the apatite structure [25,31]. The IR band from 570 cm^−1^ is characteristic of the bending mode of O-P-O vibrations in the [PO_4_]^3−^ group (ν_4_). The presence of chitosan in MgHApCs layers is spectrally distinguished by the broad IR band between 1800 and 1300 cm^−1^ and the following peaks: 1617 cm^−1^ due to C=O vibrations, 1542 cm^−1^ due to N-H vibrations, and 1248 cm^−1^ due to C-N vibrations. The 1413 cm^−1^ peak appertains to the [CO_3_]^2−^ group formed in MgHapCs layers [32].

The IR spectrum of the RPMI medium is presented in Figure 1a, (red line) where can be observed: broad and strong bands characteristic to the vibrations of the O-H groups (3790–2740 cm^−1^); small peaks at 2962, 2924, and 2852 cm^−1^ belong to C-H vibrations in the -CH_3_, -CH_2_, and C-N groups; a combination of ν_3_ and ν_1_ modes of PO_4_^2−^ ions can manifest vibrations at ~2120 cm^−1^; C-O groups, centered at 1025 cm^−1^ with two small shoulders at 1170 and 1089 cm^−1^, belong to the CO_3_ group; the C=O group has a central position at 1638 cm^−1^ and two peaks at 1729 and 1468 cm^−1^; and the unique combination of atoms in the glucose structure [33,34] possibly overlapping with the P-O vibrations in PO_4_^2−^ ions are manifested by two peaks at 601 and 560 cm^−1^ [32].

After the exposure of MgHApCs layers to electron beams at 8 and 30 Gy doses, the FTIR analysis did not reveal any major changes in the molecular structure. Similar results have been obtained previously when the irradiation of MgHApCs layers (produced in the same experimental conditions) with electron beams at 2 and 50 Gy, respectively, radiation doses did not induce detectable structural changes in the experimental FTIR spectra. Likewise, in [35], the authors reported no molecular changes in the molecular structure of HAp powders irradiated with electron beams of 1.2 MeV energy in the 1–20 Gy dose range, only recording decreases in the molecular band intensities, probably due to phosphate depletion. In our prior studies, we also identified the depletion of Ca and P in Hap-based coatings irradiated with ionizing radiation such as electron beams [25] and gamma rays [36].

In Figure 1b–d are shown the IR spectra of samples maintained in RPMI-1640 culture medium for 7 and 14 days, respectively. Molecular bands characteristic to both MgHApCs layers and the RPMI medium are identified. This indicates the embedding of the RPMI medium into the layer volume. Additionally, we suppose that the carbonation of HAp occurs as a consequence of the IR bands identified at around 1536–1545 cm^−1^, 1444–1458 cm^−1^, and 870–886 cm^−1^.

Carbonated HAp can be obtained when the carbonate ion (CO_3_^2−^) substitutes either the hydroxyl (OH^−^) A-type substitution or the phosphate (PO_4_^3−^) ion B-type substitution [32,37]. The ν_2_ vibrational mode of the carbonate group can be identified in the FTIR spectra in the wavenumber range 890–850 cm^−1^ as weak bands, while the ν_3_ vibrational mode has a characteristic wavenumber range between 1650 cm^−1^ and 1400 cm^−1^ and usually has bands with higher intensities [32,38]. IR bands at ~880 cm^−1^, ~1450 cm ^−1^, and ~1540 cm ^−1^ usually indicate A-type substitution [37,38,39], while IR bands around ~870 cm^−1^ and ~1460–1470 cm^−1^ are associated with B-type substitution [37,38,39]. AB-type substitution after the finding of ν_2_ and ν_3_ vibrational modes at 874 and 1448 cm^−1^, respectively, was also reported [37].

**Figure 2:**
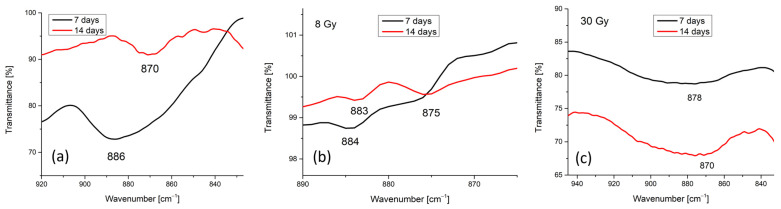
FTIR spectra in 950–800 cm^−1^: (**a**) unirradiated layers; (**b**) 8 Gy irradiated layers; (**c**) 30 Gy irradiated layers.

Therefore, we assume that the immersion for 7 and 14 days, respectively, of the unirradiated 8 Gy and 30 Gy irradiated layers in RPMI-1640 medium are conducive to B-type substitution (see Figure 2). B-type substitution is evidenced in the FTIR spectra in Figure 2, which represent the enlargement of the FTIR spectra from Figure 1 in the 950–840 cm^−1^ range. The peaks from 886, 884, and 878 cm^−1^ (see Figure 2a–c-black line) could correspond either to HPO_4_^−^ groups or to CO_3_^2−^ groups. The presence of ~880 cm^−1^ peaks in both irradiated and unirradiated samples was evidenced in our previous study [25] in the deconvoluted spectra of MgHApCs layers exposed and unexposed to 2 and 50 Gy radiation doses of the electron beams. As the FTIR spectra from Figure 1a (black line) are similar to the ones in Figure 8d,f,h of [25], we suppose that these peaks were present in the samples before their immersion in RPMI-1640 medium.

B-type substitution identified by FTIR spectroscopy analysis indicates the formation of a HAp carbonated layer after 14 days of RPMI-1640 medium immersion. The intensities of the bands centered at 870 (Figure 2a,c) and 875 cm^−1^ (Figure 2b), respectively, are almost the same, so it can be concluded that a layer of almost the same thickness is grown on all the analyzed samples. That said, it needs to be specified that the immersed samples are kept in RPMI-1640 medium at 37 °C temperature and, therefore, A-type substitution is not possible as it is usually produced at 800–1000 °C temperatures in a dry CO_2_ atmosphere [40]. Only B-type substitution takes place in wet atmospheres at temperatures ranging between 20 and 120 °C by hydrolysis reactions [40].

### 3.3. SEM and EDS Analysis

The morphological features of the surfaces of the MgHApCs layers exposed and unexposed to 8 and 30 Gy electron beam radiation doses, kept in the RPMI-1640 medium, are investigated.

**Figure 3:**
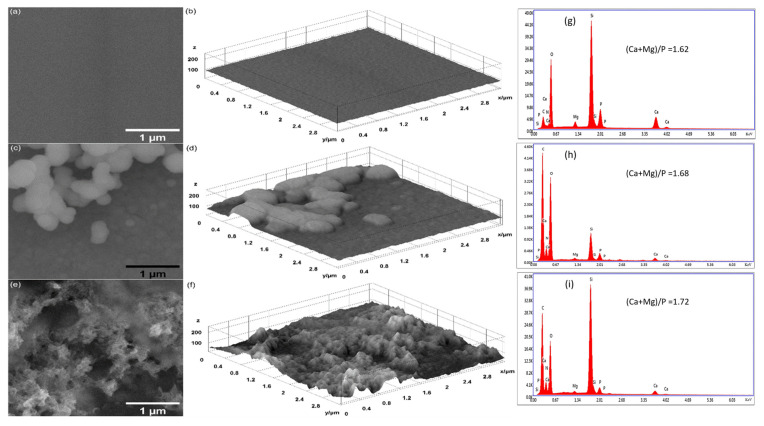
SEM images: (**a**) MgHApCs, (**c**) MgHApCs-7D, and (**e**) MgHApCs-14D layers. Three-dimensional surface plot of SEM images: (**b**) MgHApCs, (**d**) MgHApCs-7D, and (**f**) MgHApCs-14D layers. EDS spectra: (**g**) MgHApCs, (**h**) MgHApCs-7D, and (**i**) MgHApCs-14D layers.

The surfaces of unirradiated layers are smooth and uncracked before and after their keeping in cell culture medium, as is shown in Figure 3a,b. After 7 days in the RPMI-1640 medium, the layer surfaces begin to develop spherical structures in a non-uniform manner. Some of them are embedded in the layers, while others agglomerate. After 14 days, on the surface of the layer, some dense structures grow. Their formation can be connected to the increase in the (Ca + Mg)/P atomic ratio (see Figure 3g–i), which is proven to depend on the immersion duration time of the layers in the RPMI-1640 medium. It has been reported that the growth of a carbonated layer on the surface of calcium phosphates kept in biological media leads to higher Ca/P atomic ratios when CO_3_^2−^ ions are substituted for the PO_4_^3−^ ions [35,41].

**Figure 4:**
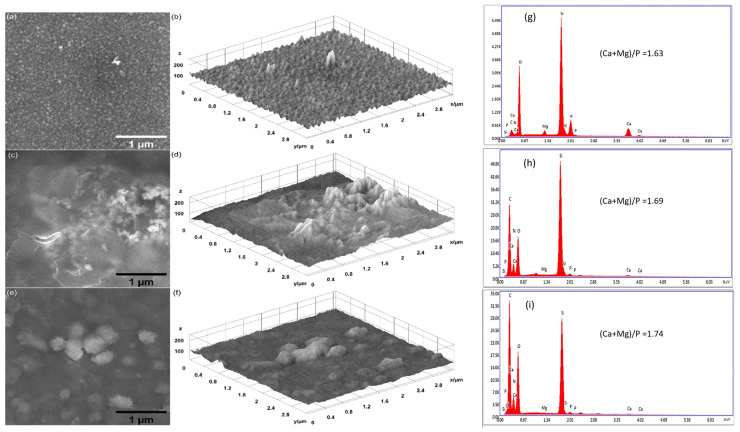
SEM images: (**a**) MgHApCs-8Gy, (**c**) MgHApCs-8Gy-7D, and (**e**) MgHApCs-8Gy-14D layers. Three-dimensional surface plot of SEM images: (**b**) MgHApCs-8Gy, (**d**) MgHApCs-8Gy-7D, and (**f**) MgHApCs-8Gy-14D layers. EDS spectra: (**g**) MgHApCs-8Gy, (**h**) MgHApCs-8Gy-7D, and (**i**) MgHApCs-8Gy-14D layers.

**Figure 5:**
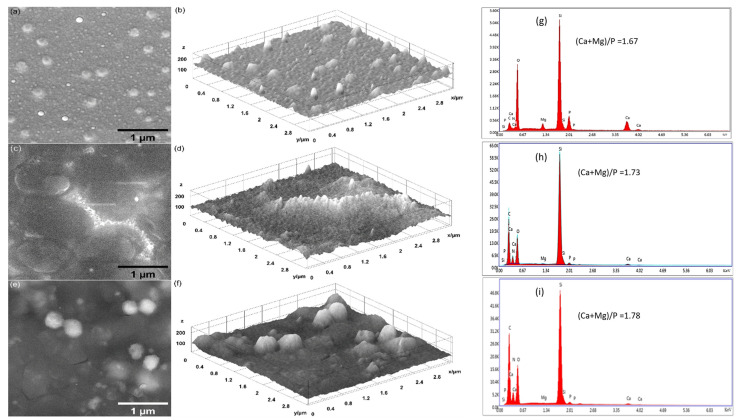
SEM images: (**a**) MgHApCs-30Gy, (**c**) MgHApCs-30Gy-7D, and (**e**) MgHApCs-30Gy-14D layers. Three-dimensional surface plot of SEM images: (**b**) MgHApCs-30Gy, (**d**) MgHApCs-30Gy-7D, (**f**) MgHApCs-30Gy-14D layers. EDS spectra: (**g**) MgHApCs-30Gy, (**h**) MgHApCs-30Gy-7D, and (**i**) MgHApCs-30Gy-14D layers.

SEM images of the surfaces of MgHApCs layers after their exposure to 8 and 30 Gy radiation doses are presented in Figure 4 and Figure 5. The presence of the polymer in the layers is conducive to surface nano-sized patterning and shrinkages, in accordance with our previous studies [25]. After 8 Gy exposure, uniform shapes of 60 nm in diameter are identified (see Figure 4a). In the case of samples irradiated with 30 Gy, the spherical-shaped structures have sizes ranging from 80 to 200 nm (see Figure 5a). In our previous work, at 50 Gy radiation dose exposure, the spherical structures were more uniform, having dimensions of about 300 nm. This is an indication that the uniform coagulation of structures into bigger ones is carried out at doses with higher values, where polymer shrinkage is advanced.

By holding the irradiated samples in the RPMI medium for 7 and 14 days, the SEM investigations show on their surfaces various types of structures that develop in time, coalescing and growing up to 1 µm in size (see Figure 4c,e and Figure 5c,e). Similar growing structures are observed in the both cases of layers irradiated with 8 and 30 Gy doses. Unfortunately, the embedding of the RPMI medium into layers exposed to electron beams produces slight cracking, as visible in Figure 4c,e and Figure 5c,e.

The surface textures of all the unirradiated and irradiated layers before and after their maintenance in RPMI-1640 medium are illustrated by 3D surface plots (see Figure 3b,d,f, Figure 4b,d,f, and Figure 5b,d,f) of the SEM images presented in Figure 3, Figure 4 and Figure 5.

The EDS spectra of MgHApCs irradiated layers (see Figure 4g and Figure 5g) in comparison with the unirradiated ones (see Figure 3g) indicated small amounts of C, O, and N atoms, which can be explained by layer shrinkage due to enhanced crosslinking and polymer depletion. Otherwise, in all the RPMI-1640-immersed samples, supplementary amounts of C and O were revealed by the EDS spectra in Figure 3h,i, Figure 4h,i, and Figure 5h,i. This indicated the absorption in the layers of the compounds contained in the RPMI-1640 medium such as sodium bicarbonate, glucose, or phenol red. The (Ca + Mg)/P atomic ratios also increased with the duration of layer maintenance in RPMI-1640 medium. In [37], it was reported that the increase in the Ca/P ratio is associated with B-type substitution and with the growth of carbonated HAp layers on HAp compounds immersed in biological media. Therefore, in agreement with FTIR spectra analysis and early research, the formation of a new carbonated HAp layer was confirmed. Previously, in [26,37,38,39,40], carbonated layers were observed only in calcium phosphate layers unexposed to ionizing radiation.

Besides Ca, P, Mg, O, C, and N atoms, the EDS spectra of all analyzed layers do not reveal other chemical elements to be an indication of the clean media favorable to cell culture growth.

### 3.4. Biological Evaluation of the Layers

**Figure 6:**
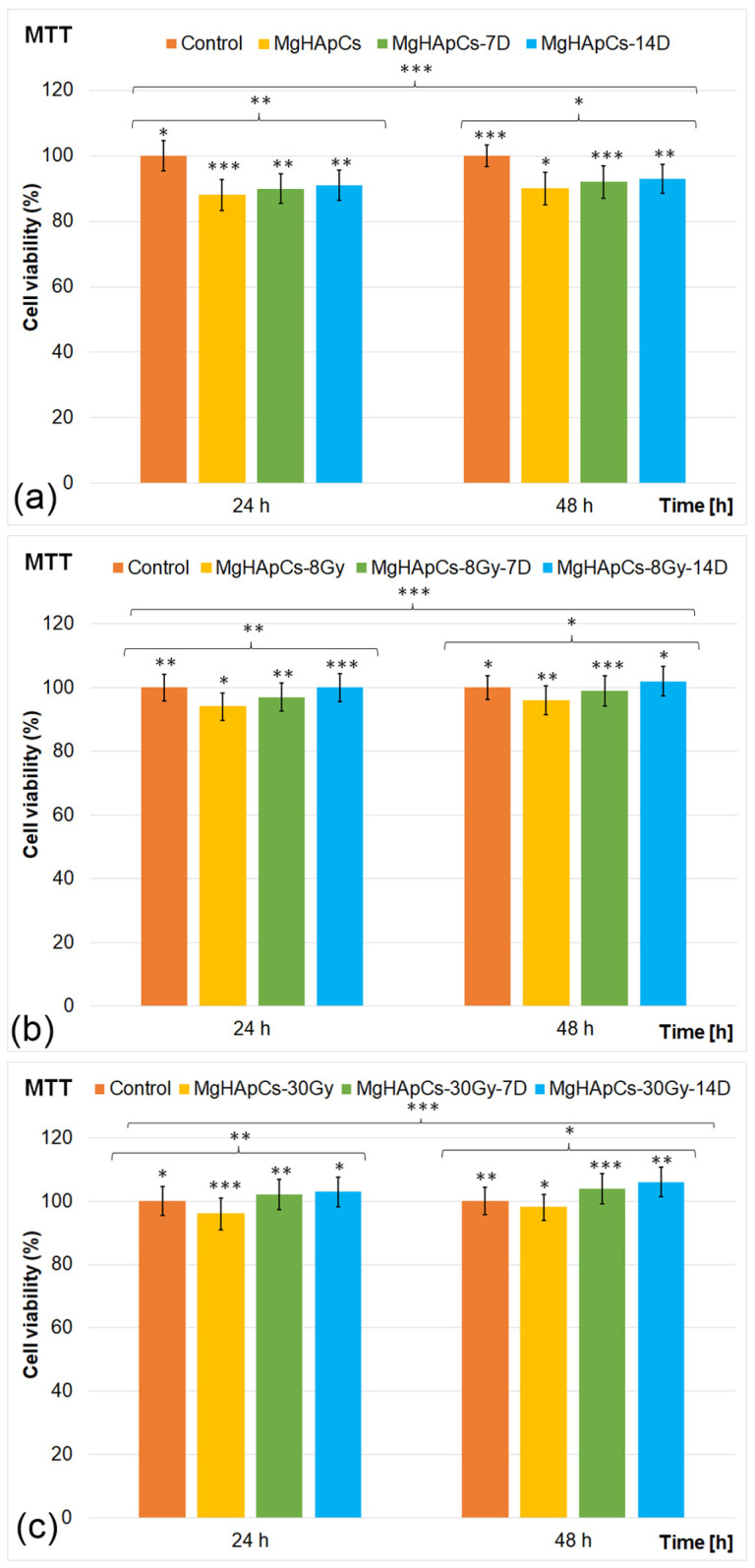
MTT assay of MG63 cells incubated for 24 and 48 h: (**a**) MgHApCs layers unirradiated and immersed in RPMI for 7 and 14 days; (**b**) MgHApCs layers irradiated with 8 Gy and immersed in RPMI for 7 and 14 days; (**c**) MgHApCs layers irradiated with 30 Gy and immersed in RPMI for 7 and 14 days. The results are represented as mean ± standard deviation (SD) and are expressed as percentages of control (100% viability). The statistical analysis was conducted using one-way ANOVA. The *p*-values indicated are the following: * *p* ≤ 0.03, ** *p* ≤ 0.002, and *** *p* ≤ 0.001.

The biological properties of the MgHApCs layers unirradiated and irradiated using 8 and 30 Gy doses before and after being immersed in RPMI-1640 medium for 7 and 14 days were investigated through in vitro studies using the MG63 human osteosarcoma cell line. The in vitro assays were conducted in triplicate, and the cell viabilities of MG63 cells incubated with the layers were evaluated after 24 and 48 h of incubation. The MTT assay results are presented in Figure 6a–c. An untreated MG63 cell culture was used as the control group. The results of the MTT assay demonstrated that the MgHApCs layers exhibited excellent biocompatibility compared to the control group after 24 and 48 h of incubation. The results also highlighted that the cell viability of MG63 cells was influenced by the irradiation dose applied to the MgHApCs layers, the incubation time, and the time that the layers were exposed to RPMI-1640 medium. The viability assays showed that the MG63 cells’ viability when incubated with MgHApCs unirradiated layers was above 88% after 24 h of incubation and increased to 90% after 48 h of incubation. More than that, the results showed that the immersion of the layers in RPMI-1640 medium for 7 and 14 days influenced, in a positive way, the layer’s cytotoxicity. The cell viabilities of MG63 cells incubated with MgHApCs immersed in RPMI-1640 for 7 days and 14 days reached 92% and 93%, respectively. More than that, the results also showed that the MG63 cells’ viability exceeded 94% for MgHApCs-8Gy and 96% for MgHApCs-30Gy after 24 h of incubation, showing an increasing trend in correlation with the irradiation dose applied to the layers. Furthermore, the data revealed that cell viability reached approximately 96% for the sample irradiated with a dose of 8 Gy and 98% for the sample irradiated with a dose of 30 Gy after 48 h of incubation. On the other hand, the findings of the MTT assays emphasized that the MgHApCs-8Gy and MgHApCs-30Gy layers immersed in RPMI-1640 medium for 7 and 14 days exhibited increased cell viability, reaching 102% and 106%, respectively, after 48 h of incubation.

The MTT assay’s results demonstrated the influence of the RPMI-1640 medium on the biological properties of both unirradiated and irradiated MgHApCs layers. The RPMI medium, which contains essential nutrients, amino acids, and vitamins, plays a critical role in maintaining the metabolic activity and proliferation of MG63 cells. When MgHApCs thin films were immersed in RPMI-1640 medium, no cytotoxic effects were observed, as indicated by the high cell viability results across all tested samples. This suggests that the interaction between the composite layers and the RPMI-1640 medium did not release any toxic by-products that could exhibit any toxic effects on cell growth. Instead, the results showed that immersion in RPMI-1640 could facilitate the obtaining of an optimal environment for cell attachment and proliferation, potentially contributing to the observed increase in viability, particularly with higher irradiation doses. These findings indicated that all tested MgHApCs composite layers maintained high cell viability for MG63 cells after 24 and 48 h of incubation. Furthermore, the results suggested that irradiation positively influenced MG63 cell viability. Additionally, immersion in RPMI-1640 medium supported cell health and metabolic activity, further reinforcing the biocompatibility of the composite thin films. The results are in good agreement with previously reported studies regarding the influence of gamma irradiation and biological medium immersion on the physicochemical and biological properties of hydroxyapatite composite layers [26,28,42,43,44,45,46,47].

**Figure 7:**
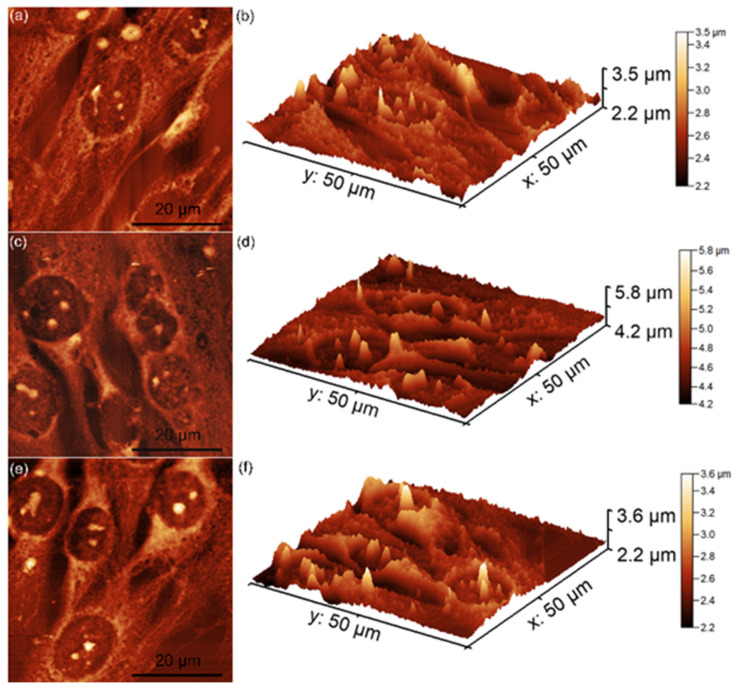
Two-dimensional AFM topographies of MG63 cells after 48 h of incubation with MgHApCs unirradiated layers (**a**) and immersed in RPMI for 7 days (**c**) and 14 days (**e**), as well as their 3D representations (**b**,**d**,**f**).

**Figure 8:**
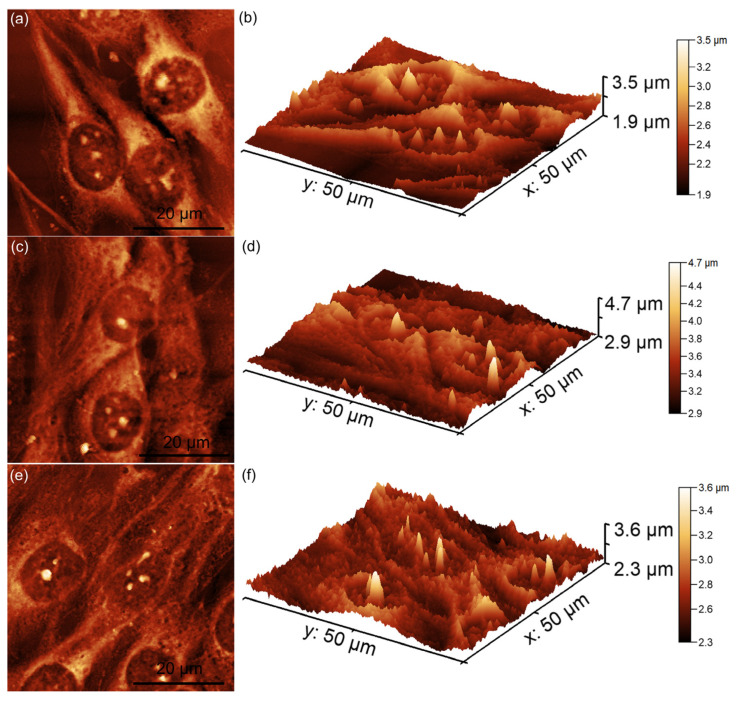
Two-dimensional AFM topographies of MG63 cells after 48 h of incubation with MgHApCs layers irradiated with 8 Gy (**a**) and immersed in RPMI for 7 days (**c**) and 14 days (**e**), as well as their 3D representations (**b**,**d**,**f**).

**Figure 9:**
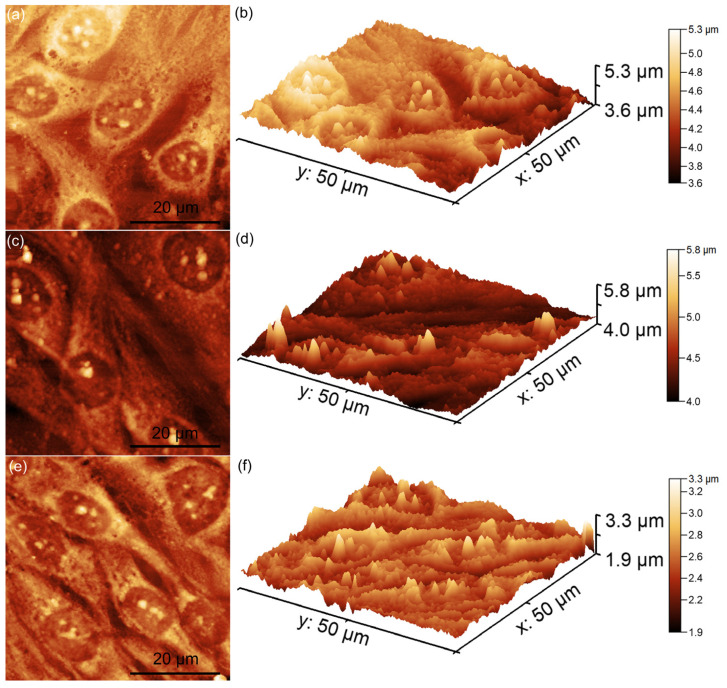
Two-dimensional AFM topographies of MG63 cells after 48 h of incubation with MgHApCs layers irradiated with 30 Gy (**a**) and immersed in RPMI for 7 days (**c**) and 14 days (**e**), as well as their 3D representations (**b**,**d**,**f**).

Additional information about the attachment and proliferation of MG63 cells after 48 h of contact with unirradiated and irradiated MgHApCs layers (8 Gy and 30 Gy) immersed in RPMI-1640 medium for 7 and 14 days was also obtained using AFM. For this purpose, all the MgHApCs layers were incubated with MG63 cell suspensions for 48 h, after which the cells were fixed on the layer surfaces and analyzed by AFM in non-contact mode under normal atmospheric conditions. The AFM topographies were recorded on a 50 × 50 μm^2^ surface area, and the results are presented in Figure 7, Figure 8 and Figure 9.

As demonstrated in previous studies by Knápek et al. [48,49], AFM non-contact mode allows reliable and non-destructive surface analysis. The 2D AFM topographies revealed that MG63 cells adhered to all tested surfaces after 48 h of incubation. However, some minor morphological differences were observed based on the irradiation dose. On the unirradiated and 8 Gy-irradiated MgHApCs layers, the cells displayed elongated and well-oriented morphologies, indicative of healthy fibroblastic-like MG63 cells. These findings suggest that the surfaces provided favorable conditions for MG63 cell attachment and development (Figure 8a–f).

Furthermore, in the case of the 30 Gy-irradiated MgHApCs layers, the MG63 cells exhibited slight morphological alterations, including a rounder and more isolated appearance, which deviates from the typical elongated MG63 morphology (Figure 9a–f). This might indicate that a higher irradiation dose may have influenced the biocompatibility of the composite layers, affecting cell attachment and development. The results of the 2D AFM topographies were further corroborated by their corresponding 3D representations. Both 2D and 3D images confirmed that unirradiated and 8 Gy-irradiated MgHApCs layers supported MG63 cell attachment and proliferation, forming well-aligned monolayers with fibroblastic morphology. However, the 30 Gy-irradiated surfaces induced minor changes in cellular morphology, suggesting a dose-dependent effect on cell compatibility. These findings are consistent with previously reported studies on the influence of irradiation on the biocompatibility of magnesium-doped hydroxyapatite/chitosan composite layers [50,51]. Furthermore, the data also suggested that the immersion of MgHApCs layers for 7 and 14 days in RPMI-1640 medium significantly influenced MG63 cell adherence and development. These results could be attributed to the fact that RPMI-1640 is enriched with essential nutrients and supplements, which might help to enhance the cell viability, attachment, and proliferation of MG63 cells on the surfaces of MgHApCs layers.

There are several literature studies that report on the significant influence of biological mediums on the surface properties of HAp coatings, which are widely used in biomedical applications such as bone implants and tissue engineering [26,43,46,47,52]. This behavior is attributed to the fact that, when exposed to biological environments such as simulated body fluid (SBF), blood plasma, or other cell culture mediums, HAp coatings often undergo surface modifications, including ion exchange and dissolution–reprecipitation, leading to the formation of additional calcium phosphate phases [26,43,46,47,52]. Reports also indicate that proteins and other biomolecules present in biological mediums can adsorb onto the HAp surface, further impacting its biocompatibility and cell adhesion properties. Such studies underscore the importance of understanding the interactions between HAp coatings and biological mediums to optimize their performance in clinical applications. The AFM analysis showed that the MG63 cells cultured on MgHApCs layers immersed for 7 and 14 days in RPMI-1640 medium exhibited well-organized, elongated fibroblastic morphologies typical of normal healthy cells. More than that, the AFM 2D topographies, as well as their 3D representations, emphasized that the cells formed monolayers, which is a sign of healthy cell growth. In contrast, the MG63 cells cultured on the MgHApCs layers that were not immersed in RPMI-1640 exhibited less consistent attachments, having fewer well-aligned cells and also showing lower proliferation rates. These findings are in good agreement with the MTT results and emphasize the significant influence of the RPMI-1640 medium in improving the biocompatibility of MgHApCs layers, likely due to its balanced composition of amino acids, vitamins, and glucose, which are good supports for the cell’s adhesion and growth.

## 4. Conclusions

Magnesium-doped hydroxyapatite/chitosan composite coatings covered Si substrates with mirror-like surfaces after their synthesis by the radio-frequency magnetron sputtering technique. The exposure of the layers to electron beams of 5 MeV in energy and 8 and 30 Gy radiation doses, respectively, was conducive mainly to morphological modifications of the layer surfaces rather than of their molecular structure. Mechanical measurements indicated that the values of Young’s modulus and stiffness decrease as the irradiation dose increases, most probably due to chitosan depletion. These experimental data are sustained by the morphological features of the analyzed samples, which show nano-sized patterning of the layer surfaces due to polymer shrinkage as the value of the ionizing radiation dose increases. Consecutively, the arithmetic mean deviation Ra of the roughness profile values increases.

Changes in the molecular structure of the irradiated and unirradiated samples were observed by FTIR spectroscopy after their immersion in RPMI-1640 cell culture medium for 7 and 14 days. The presence of the ~870 cm^−1^ peak in the FTIR spectra of the layers indicated the substitution of PO_4_^3−^ groups from the hydroxyapatite structure with CO_3_^2−^ groups from the biological medium, which is characteristic of B-type substitution in the hydroxyapatite structure. The formation of the carbonated HAp layer was also sustained by the increase in the (Ca + Mg)/P ratio with the duration of time spent keeping the samples in the biological medium, as the EDS analysis reveals.

The results of the biological assays demonstrated the significant role of MgHApCs composite layers in supporting MG63 cell adherence and proliferation. Furthermore, the findings highlighted that the biological properties of the MgHApCs layers were significantly influenced by both immersion in RPMI-1640 medium and the irradiation doses. The AFM analyses revealed that the unirradiated layers and 8 Gy-irradiated MgHApCs layers provided good conditions for MG63 cell attachment, promoting a well-organized, elongated fibroblastic cell morphology. However, the MgHApCs layers irradiated using higher irradiation doses, such as 30 Gy, led to minor morphological alterations in MG63 cells, suggesting that the irradiation dose influenced the layers’ biocompatibility. Additionally, the data also revealed that immersion in RPMI-1640 medium helped to enhance the biocompatibility of MgHApCs layers, improving MG63 cell attachment and proliferation. These findings emphasize the potential of MgHApCs composite layers as promising biomaterials for the development of future biomedical applications while highlighting the importance of irradiation parameters and culture conditions in influencing cell behavior.

## Figures and Tables

**Table 1:** Mechanical results of MgHApCs layers exposed and unexposed to electron beam irradiation.

Samples	R_a_ (µm)	Young’s Modulus (GPa)	Stifness (µN/nm)
MgHApCs layer	0.010 ± 0.001 (0.1%)	58.41 ± 5 (0.08%)	10 ± 1 (0.1%)
MgHApCs-8Gy layer	0.011 ± 0.001 (0.1%)	31.95 ± 2 (0.06%)	9 ± 0.9 (0.1%)
MgHApCs-30Gy layer	0.016 ± 0.001(0.1%)	5.19 ± 0.05 (0.01%)	2.2 ± 0.02 (0.1%)

## Data Availability

The data presented in this study are available on request from the corresponding author.
